# Midgut serine proteases and alternative host plant utilization in *Pieris brassicae* L.

**DOI:** 10.3389/fphys.2015.00095

**Published:** 2015-03-31

**Authors:** Rakesh Kumar, Usha Bhardwaj, Pawan Kumar, Sudeshna Mazumdar-Leighton

**Affiliations:** Insect-Plant Interactions Group, Department of Botany, Delhi UniversityDelhi, India

**Keywords:** lepidoptera, digestive physiology, serine proteases, plant protease inhibitors, nutrition

## Abstract

*Pieris brassicae* L. is a serious pest of cultivated crucifers in several parts of the world. Larvae of *P. brassicae* also feed prolifically on garden nasturtium (*Tropaeolum majus* L., of the family Tropaeolaceae). Proteolytic digestion was studied in larvae feeding on multiple hosts. Fourth instars were collected from cauliflower fields before transfer onto detached, aerial tissues of selected host plants in the lab. Variable levels of midgut proteases were detected in larvae fed on different hosts using protein substrates (casein and recombinant RBCL cloned from cauliflower) and diagnostic, synthetic substrates. Qualitative changes in midgut trypsin activities and quantitative changes in midgut chymotrypsin activities were implicated in physiological adaptation of larvae transferred to *T. majus*. Midgut proteolytic activities were inhibited to different extents by serine protease inhibitors, including putative trypsin inhibitors isolated from herbivore-attacked and herbivore-free leaves of cauliflower (CfTI) and *T. majus* (TpTI). Transfer of larvae to *T. majus* significantly influenced feeding parameters but not necessarily when transferred to different tissues of the same host. Results obtained are relevant for devising sustainable pest management strategies, including transgenic approaches using genes encoding plant protease inhibitors.

## Introduction

*P. brassicae* (the Large Cabbage Butterfly) of the order Pieridae is purported to have a Palearctic distribution with reports from Asia, Europe, and North Africa of host plants belonging to families Cruciferae, Resedaceae, Papilionaceae, Umbelliferae, and Trapaeolaceae (Feltwell, 1978). Choice and performance of pierid insects on different host plants have been extensively investigated by several groups in Europe and America (Schoonhoven et al., 2007). In North India, *P. brassicae* is a recurrent, euryophagous (broad host range) pest of cultivated winter crops like cauliflower, cabbage, mustards, and radish (Hussain, 1924; Kaushal and Vats, 1983; Lal and Ram, 2004; Ali and Rizvi, 2007; Hasan and Ansari, 2010; Kular and Kumar, 2011). Broods of *P. brassicae* frequently defoliate host plants and cause farmers to spray insecticides including highly hazardous class 1b organo-phosphates (Sharma and Gupta, 2009; Weinberger and Srinivasan, 2009). Farms in the region are generally small-holdings where different crucifers are planted in adjacent fields as winter crops (Weinberger and Srinivasan, 2009). In years of high pest density, *P. brassicae* larvae are highly mobile and move from an exhausted food source to a proximal, alternative host plant of the same or different species. Larvae have been reported to move from and between various crucifers (Chew, 1980; Davies and Gilbert, 1985; Le Masurier, 1994; Muriel and Grez, 2002; Lucas-Barbosa et al., 2014).

An alternative host species of *P. brassicae* in North India is the garden nasturtium or *Tropaeolum majus* (Dhiman et al., 2009; Kumar, 2009). *T. majus* occurs in cultivated gardens as well as feral patches in North India (Babu, 1977). There is some controversy in the literature about *T. majus* as a host of pierid larvae. While some reports cite the inability of larvae to feed on *T. majus*, (Huang and Renwick, 1995; Renwick and Huang, 1995), others cite the inability of larvae “habituated” on crucifers to feed on *T. majus* and *vice versa* (Hovanitz and Chang, 1962, 1963; Ma, 1972; Kaushal and Vats, 1983; Rotem et al., 2003). Not much is known about the digestive physiology of *P. brassicae* with respect to performance on different species of host plants in North India. Physiological adaptations enabling euryophagy and the effects of diet shift on gut proteases, feeding parameters/nutritional indices of *P. brassicae* larvae are not well understood.

Like other Lepidoptera, digestive serine proteases have been reported from various pierid larvae (Broadway, 1989a; Broadway and Colvin, 1992; Liao et al., 2007; Zhou et al., 2008; Zibaee, 2012; Bhardwaj et al., 2014). Midgut trypsins and chymotrypsins can digest ingested plant tissues containing proteins (like Rubisco) that are sources of amino acids and energy essential for insect growth and development (Christeller et al., 1992; Woods and Kingsolver, 1999). Ingested plant tissues are complex and include antifeedants like plant protease inhibitors (PPIs) that can rapidly alter expression levels of serine proteases produced in the lepidopteran larval midgut (Broadway, 1995, 1996; Jongsma et al., 1995; Telang et al., 2005; Terra and Ferreira, 2012). Ingestion of cabbage protease inhibitors is known to alter expression of midgut trypsin and chymotrypsin activities in *P. rapae* (Broadway and Colvin, 1992). Physiological responses elicited in actively feeding larvae reflect variation in time and space of ingested defense compounds and nutritional quality of host plant tissues (Broadway and Duffey, 1986a,b; Geiselhardt et al., 2013). Expression of inhibitor-insensitive proteases in several lepidopteran larvae involves complex, transcriptional responses that occur within hours of exposure to an ingested, heterologous PI (Broadway, 1996; Mazumdar-Leighton and Broadway, 2001b; Volpicella et al., 2003; Vogel et al., 2014). Plant protease inhibitors when ingested along with secondary metabolites and plant defense compounds induce complex transcriptomic and proteomic responses in the lepidopteran gut associated with herbivory (Vogel et al., 2014).

*P. brassicae* utilize glucosinolates (like sinigrin) present in host plant foliage as oviposition cues and phagostimulants. Larval midgut proteins like the nitrile-specifier proteins (NSP) detoxify ingested reactive nitriles produced by the host plant Glucosinolate—Myrosinase systems (Wittstock et al., 2004; Stauber et al., 2012). Hence, both NSP production and PPI-insensitive proteases are likely adaptive responses occurring in Pierids larvae during herbivory (Agrawal, 2000). Life history traits of Lepidoptera have been shown to be influenced by the choice of host plant (Mattiacci et al., 2001; Agrawal et al., 2002; Metspalu et al., 2003; Rotem et al., 2003; Hasan and Ansari, 2011; Zibaee, 2012; Paz Celorio-Mancera et al., 2013). Long and short term adaptive physiological responses and associated costs can potentially alter life histories and genetic fitness in the lepidopteran larvae (Rausher, 1982; Gotthard et al., 1994) Fitness costs are likely associated with physiological plasticity exhibited by the lepidopteran digestive system when larvae feed on different diets (Mattiacci et al., 2001; Agrawal et al., 2002; Cipollini et al., 2003; Paz Celorio-Mancera et al., 2013).

This paper examines if, and how levels of midgut proteases differ in larvae feeding on different hosts. Implications for sustainable strategies of managing the pest in the region including transgenic approaches with protease inhibitors are discussed.

## Materials and methods

### Insect and plant materials

Farmer fields in village Kabirpur, Sonepat district in Haryana, North India, lying between 28° 59′ 0″ North, 77° 1′ 0” East were used for collection of crucifer plant and insect materials. GPS co-ordinates were determined using a Garmin eTrex Vista instrument according to instructions provided by the vendor (Garmin Inc., KS, USA). Egg clusters in 2 ha^2^ fields were carefully monitored between the months of September-November. Freshly molted, healthy fourth instar larvae from at least three independent egg clusters of *P. brassicae* (*n* = 300 ± 20), feeding on mature leaves (ML) of 40-day old cauliflower, *Brassica oleracea* var. *botrytis* cv. Madhuri were collected in the month of October. Insects were identified at the Indian Agricultural Research Institute, New Delhi, India. Larvae feeding on mature leaves of cabbage, *B. oleracea* var. *oleracea* cv. Golden Acre; mustard, *B. campestris* var. *sarson* cv. peeli sarson, and radish, *Raphanus sativa* cv. Pusa Desi were sampled simultaneously from neighboring fields cultivated under similar edaphic conditions. Standard procedures for cultivation, including tube well irrigation, rotation with wheat/rice, use of NPK fertilizer (150 kg N, 120 kg P_2_O_5_, and 60 kg K per ha) were adopted. Fields earmarked for collection of insect and plant material were not sprayed with insecticides at the time of collection. Larvae found feeding on leaves of feral patches of *T. majus* that were flowering in cultivated gardens patches at Delhi University were sampled similarly. Head capsule sizes of fourth instar larvae feeding on cauliflower was approximately 0.204 ± 0.001 cm (*n* = 100).

### Insect and plant materials for “transfer” experiments to different host plants

Field-collected fourth instar larvae (henceforth referred to as “control or ctrl”) were immediately processed for protease assays as described below. For “transfer” experiments, fourth instars were collected from synchronized egg clusters found feeding on mature leaves of cauliflower plants. These larvae were brought to the lab within an hour of collection along with the plant tissues on which they were feeding and placed in plastic cups (12 × 8 × 5 cm). Larvae were starved to empty gut contents and randomly divided into batches of 4 larvae per replicate. Since *P. brassicae* is a gregarious feeder, four larvae were used per replicate. At least three biological replicates (12 × 3 = 36 larvae) were gently air brushed onto hydrated, plant tissues being tested and allowed to feed for 12 h. In addition to the single tissue preferred by the larvae (= from which the larvae had been captured in the field), additional plant tissues available simultaneously were also tested. These included young leaf (YL) and curd or head (HD) tissues for cauliflower. Mature leaves on 40–45 days old cauliflower plants were fully expanded, dark green, large, and usually those oviposited on; young leaves were smaller, light green in color while head was the future inflorescence developing into fruit. Mature leaves comprised the middle whorls of each plant; younger leaves comprised the innermost whorls immediately adjacent to the developing curd/head. Senescent leaves were not used. Plants used here corresponded to principal growth stage 4 (development of harvestable vegetable plant parts) in cauliflowers (Feller et al., 1995). Density (g/m^2^) and specific leaf weight or SLW (g dry wt/m^2^) were recorded for each plant tissues (Waller and Jones, 1989). Corresponding estimates from intact plants sampled from the field were the highest in the intact mature leaf tissues, followed by young leaf tissues and the head tissues (Kumar, 2009). Leaf area consumed was equivalent to biomass removed. As the duration of the feeding experiment was short (12 h), the insect probably used volumetric regulation for meal size. For *T. majus* the tissues examined were leaf (L) and flower (F). No distinction (viz. young/mature) was possible between the fully expanded leaves of 60–65 days old *T. majus*, as larvae were found to feed prolifically on all available foliar tissues (Leaf diameter = 3.32 ± 0.13 cm, *n* = 25).

### Feeding assays for larvae reared on cauliflower and transferred to different host plants

No-choice experiments were performed (in preference over binary choice) as they are generally held to be more representative of the field situation in agricultural systems (Manly, 1995; Bernays and Weiss, 1996). The nomenclature used in this study was as follows: fourth instar larvae collected from cauliflower (CF) ML were transferred in the lab to YL, ML and HD tissues of cauliflower (CF-CF YL, CF-CF-ML, CF-CF-HD); L of *T. majus* (CF-TP-L), and F of *T. majus* (CF-TP-F). Transferred larvae were allowed to feed for 12 h duration as it represented about 1/3rd time taken by this larval phase (Kumar, 2009). Larvae were always provided with equivalent field-collected fresh deveined plant material in case the food was consumed. For each tissue type evaluated, feeding parameters, nutritional indices and midgut protease activities were determined. The amount of leaf ingested (in gm), fecal matter produced (in gm), and larval weight increases were recorded for 12 h from each plant type and tissue being examined using micro-weighing balance (0.001 decimal; Sartorius AG, Goettingen, Germany). Dry weights were extrapolated from three replicates of ten fourth instar larvae and 10 plant tissues of equivalent size and age heated in an oven (Elektro-Helios, Stockholm, Sweden) set at 65°C until complete dryness. Correction factors for water loss, different metabolism of plant tissue types following detachment from plant and different contribution of gut contents (Bowers et al., 1991) were taken into account during measurement of nutritional indices.

The various nutritional indices measured according to standard protocols (Waldbauer, 1968; Slansky and Scriber, 1985) were as follows: Approximate Digestibility (AD): (dry weight of food ingested—dry weight of feces/dry weight of food ingested) × 100; Efficiency of conversion (ECI) of ingested food to body matter: (Weight gained/dry weight of food ingested) × 100; Efficiency with which digested food is converted to body matter (ECD): (weight gained/dry weight of food ingested—dry weight of feces) × 100. Midgut samples were also obtained from larvae collected from cauliflower field as neonates, reared on cauliflower ML in the lab and transferred as fourth instars (after overnight starvation) to various tissues of cauliflower in no-choice, fixed time feeding experiments as described above. This was done as larvae reared on intact plants can perform differently when compared to larvae fed detached leaf tissues (Rayapuram and Baldwin, 2006). Conditions for lab-rearing of insects were 25°C ± 3, 70% relative humidity and 16:8 light-dark regimes. In the above experiments, insects were used immediately after molting as larval feeding rates increase significantly after each molt (Farrar et al., 1989). All samples were evaluated simultaneously. Larvae were then degutted, carcass used for estimating dry weight gut contents (Bowers et al., 1991) and the midguts isolated for protease assays.

### Detection of midgut proteases using protein substrates and gelatin zymograms

Total midgut protease activity was measured in field-collected fourth instar larvae feeding on mature leaves (ML) of cauliflower, cabbage, mustard, radish and *T. majus*. Rubiscolytic activities were detected using purified Rubisco from *N. tabacum* foliage after Bhardwaj et al. (2014). Briefly, 1 μg protein was incubated with 200 μg of purified Rubisco in 100 mM Glycine-NaOH buffer, pH 10. Undigested protein substrate was precipitated by adding 1.3 ml of 10% trichloroacetic acid (TCA; catalog # 204842, SRL Pvt. Ltd., Delhi, India; w/v) and the sample mixture was incubated at 4°C for 15 min. The samples were centrifuged at 13,000 rpm for 15 min at room temperature. TCA soluble peptides in the supernatant were measured at 280 nm using UV Spectrophotometer (UV VIS Spectrophotometer 119, Systronic Inc., India). Recombinant RBCL (rRBCL-Cf) was obtained by cloning the *rbcL* gene fragment amplified from cauliflower (GenBank accession # EU128739) and expressing it in pET expression system in *E. coli* (Rani, 2012). Following purification and refolding from inclusion bodies, the recombinant protein was used along with casein in a standard TCA precipitation assay as mentioned earlier. Assays were performed with or without various protease inhibitors in the assay buffers (pH 9 and 10). Suitable blanks were included to measure endogenous digestion of the protein substrates in absence of enzyme. Enzyme only controls (without substrate), and enzyme and inhibitor controls (without substrate) were also included. Gelatinolytic zymograms were run according to the modified method of Lantz and Ciborowski (1994); Michaud (1998); Oppert et al. (1998) and Bhardwaj et al. (2014). Equal amounts of midgut extract from 4th instar larvae were applied to each lane of the activity gels.

### Assays for midgut proteases and their inhibition using synthetic substrates

*In vitro* assays for trypsin-like activity and its inhibition by soybean trypsin inhibitor kunitz (STI, catalog # T-9128, Sigma-Aldrich Inc., Saint Louis, MO, USA) were performed with the esterolytic substrate, α-Tosyl-Arginine methyl ester (TAME, catalog # T-4626, Sigma-Aldrich Inc., MO, USA) after Walsh and Wilcox (1970); and amidolytic substrate, N-α benzoyl-L-Arginine-*p*-nitroanilide hydrochloride (BApNA, catalog # B-4875, Sigma-Aldrich), after Broadway (1997). Midgut extracts were also analyzed using fluorogenic substrates: N-α benzoyl-arginine amido methyl coumarin, (BAAMC, catalog # B-7260, Sigma-Aldrich) for trypsin activity; N-α succinyl-Alanine-Alanine-Proline-Phenylalanine-amido methyl coumarin, (SAAPFAMC, catalog # S-9761, Sigma-Aldrich) and N-α succinyl-Leucine Leucine Valine Tyrosine-amido methyl coumarin (SLLVYAMC, catalog # S-6510, Sigma-Aldrich) for chymotrypsin activity and N-methoxy succinyl-Alanine-Alanine-Proline-Valine-amido methyl coumarin (MeOSuc-AAPV-MCA, catalog # BML-P224-0005, BioMol International, Plymouth Meeting, PA, USA) for elastase activity. Assays were performed in 100 mM Tris-HCl buffer, pH 8 with the TAME substrate; 100 mM Tris-HCl buffer, pH 9 for BApNA substrate, and 100 mM Glycine-NaOH buffer, pH 10 for other synthetic fluorogenic substrates.

The ability of inhibitors STI (2 mg/ml), N-α-Tosyl-L-Lysine chloromethyl ketone or TLCK (3 mg/ml, catalog # T-7254, Sigma-Aldrich), N-p-Tosyl-L-Phenylalanine chloromethyl ketone or TPCK (3 mg/ml, catalog # T-4376, Sigma-Aldrich), and Aprotinin (1 mg/ml, catalog # 10236624001, Roche Applied Biosciences, Indianapolis, IN, USA) to inhibit the serine protease activities was also investigated as described in Mazumdar-Leighton and Broadway (2001a). Assay for cathepsin-B activity was performed at pH 6.5 using SLLVYAMC as substrate and its ability to be inhibited by E-64 (1 mg/ml) (L-trans-epoxy succinylleucylamide [4-guanido] butane, catalog # E-3132, Sigma-Aldrich) was also determined. The liberation of the AMC molecules after hydrolysis of the substrates were followed using a TKO 100 mini–fluorometer (Hoefer Scientific Instruments, CA, USA) as fluorescence units released per minute.

### Isolation of putative trypsin inhibitors from cauliflower and *T. majus* leaves

About 20–25 mature leaves were collected at random from similar positions (nodes) of cauliflower and *T. majus* plants showing approximately 50% leaf area damage by *P*. *brassicae* (induced) and pooled before processing. Similar samples were collected from neighboring, insect-free, healthy plants (un-induced) during the month of October. Samples were immediately stored in liquid nitrogen. The isolation of semi-purified protease inhibitors was carried exactly after Broadway (1993). Subsequently, affinity column chromatography was performed using (bovine) trypsin-agarose (Kumar, 2009). Briefly, a fraction of ammonium sulfate precipitate showing >60% inhibition against bovine trypsin (catalog # T-1426, Sigma-Aldrich) was loaded on a trypsin-agarose (catalog # T-4019, Sigma-Aldrich) column. The inhibitor-bound column was washed extensively with 10 mM Tris-HCl, pH 8 containing 100 mM KCl until the A_280_ reached zero. Bound proteins were eluted with 8 M urea pH 3. Fractions showing >80% inhibition against 1 mg/ml bovine trypsin in BAAMC assays were pooled and dialyzed (with a cutoff of M_*r*_ 12,000) against autoclaved distilled water at 4°C overnight. Dialyzed samples were lyophilized and subjected to SDS PAGE (Laemmli, 1970). A single band of approximately 23 kD size observed for each cauliflower sample, and a single band of approximately 21 kD observed for each *T. majus* sample on 12.5% SDS PAGE gel (Kumar, 2009), was excised and processed for liquid chromatography electro-spray ionization mass spectrometry (LC-ESI-MS) as described below.

### LC-ESI-MS of CfTI, TpTI and *in vitro* inhibition assays of midgut proteases

The excised protein bands were sent to a commercial vendor, The Center for Genomic Applications (TCGA Ltd., Delhi, India). In-gel tryptic digests of the samples were subjected to two-dimensional LC-ESI-MS on an Agilent 1100 series Nano LC system coupled to an ESI-MS instrument (Agilent technologies, Santa Clara, USA). Peptides generated were processed through an ion-trap mass spectrometer and subjected to MS/MS ion searches. Data was analyzed using MASCOT search engine (http://www.matrixscience.com). Searches of MS database 20070501 were limited by taxonomy to include only Viridiplantae (Green Plants) with 247,883 sequences. Mudpit scoring was adopted to remove redundant hits of the matched peptides to homologous proteins in the database.

The putative trypsin inhibitor protein isolated from *P. brassicae*-fed (induced) cauliflower mature leaf sample will be referred henceforth as CfTI, while the putative trypsin inhibitor from *P. brassicae*-fed (induced) *T. majus* leaf sample will be referred to as TpTI. Ability of these putative trypsin inhibitors (0.8 mg/ml) to inhibit midgut proteases of 4th instar *P. brassicae* larvae collected from cauliflower ML and transferred to ML tissues of cauliflower (CF-CF-ML) and leaves of *T. majus* (CF-TP-L) were determined using BAAMC substrate at pH 10 as described earlier. For comparison STI was used at a concentration of 1 mg/ml. Controls included bovine trypsin (catalog # T-1426, Sigma-Aldrich) and bovine chymotrypsin (catalog # C-3142, Sigma-Aldrich) tested at 1 mg/ml. Enzyme activities with and without inhibitors were determined as FU released/minute/mg protein.

### Reverse zymography with purified CfTI, bovine trypsin and midgut samples

Reverse zymography using a Tris-Tricine buffer system (Le and Katunuma, 2004) was used to detect CfTI and TpTI as inhibitors of bovine trypsin. The Laemmli's buffer system with a separating gel containing gelatin (catalog # 61792405001046, Merck India Ltd., Mumbai, India) was used. Equal amount of all the samples (10 μg) were prepared without boiling in a non-denaturing loading buffer (500 mM Tris-HCl pH 6.8, 10% glycerol, 2% SDS, and 0.1% bromo-phenol blue). Along with STI, a legume trypsin inhibitor from *Mucuna pruriens* L. isolated in our lab was used as a positive control because of its ability to inhibit gut proteases of *P. brassicae* (Pant et al., unpublished). Gels were run at 120 V for about 90 min at 4°C till the dye reached the bottom of the gel. After electrophoresis, gels were washed in 2.5% Triton X-100 (catalog # T-9284, Sigma-Aldrich Inc.) for 45 min with gentle shaking to remove the SDS from the gel and to allow the enzyme to re-nature. The gels were washed 4–5 times with distilled water and kept overnight in the incubation buffer (containing proteases) at 37°C. Following incubation, gels were washed 2–3 times in distilled water and stained with 0.25% Coomassie Blue R-250 (catalog # 24018, SRL Pvt. Ltd., Delhi, India) solution. The gels were de-stained with methanol and acetic acid until fine dark bands appeared against a clear background. Total protein estimations were performed using Bradford's reagent (Bradford, 1976)

### Statistical analyses

The results of no-choice, fixed time laboratory experiments were analyzed using SPSS 14.0 (SPSS Inc., Chicago, IL, USA). Percent values of AD, ECI and ECD were arcsine transformed before analyses. The feeding parameters (plant weight/food consumed, larvae weight gain, fecal matter production), nutritional indices (AD, ECI and ECD) and enzymatic activities in *P. brassicae* midgut extracts collected from fields and transferred to alternate host plants, measured using amidolytic (BApNA), esterolytic (TAME), and fluorogenic (SAAPFAMC and BAAMC) substrates were analyzed using One-Way ANOVA by Tukey's HSD (Honestly Significant Difference) and LSD (Least Significant Difference). Since the results were similar, only results from the former statistical tests are described. The Mann Whitney test was also performed for all the above mentioned parameters for comparison between different tissues when the transfer was made to these tissues from cauliflower. Averaged results for each set of 36 larvae are described as means ± SE. Correlations between measures of feeding parameters, nutritional indices and digestive proteases were determined for all the host plant and transfers. Scatter plots were examined for Kendall Tau correlation coefficients to assess the strength of associations between these measures. Only relevant results from comparison of midgut trypsin and chymotrypsin activities with larval weight gain are described here.

## Results

### Midgut serine proteases in field-collected fourth instar larvae feeding on various crucifers and *T. majus*

**Figure 1:**
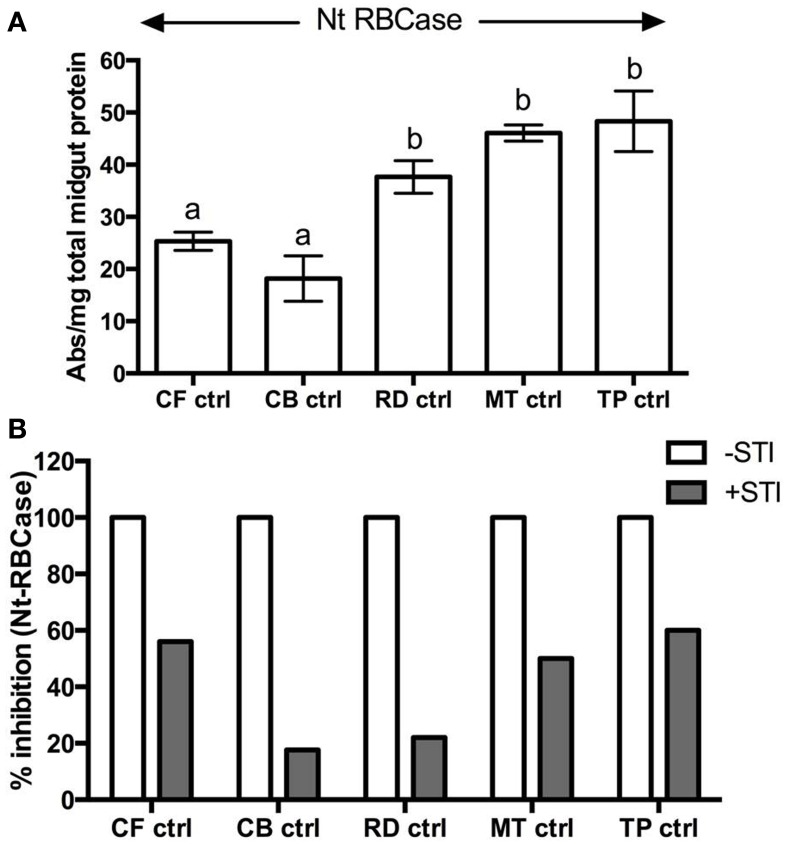
**(A)** Rubiscolytic activities detected in midgut samples (0.5 μg/μl) from field-collected, fourth instar *P. brassicae* (*n* = 36) feeding on leaves of cauliflower (CF-ctrl), *T. majus* (TP-ctrl), radish (RD-ctrl), cabbage (CB-ctrl), and mustard (MT-ctrl) using native Rubisco isolated from *N. tabacum* (Nt RBCase) as substrate. **(B)** Inhibition of rubiscolytic activities detected in the samples after incubation with STI. The extent of inhibition is shown relative to proteolytic activities detected in midgut sample (depicted as 100% in absence of STI). The midgut samples were pre-incubated with STI in 1:1 ratio (v/v) for 10 min at room temperature before adding the protein substrate.

Field-collected fourth instar larvae feeding on mature leaves of cauliflower (CF-ctrl) and cabbage (CB-ctrl) had similar levels of total midgut proteolytic activities as measured by digestion of Rubisco substrate at pH 10 (Figure 1A). Rubiscolytic activities measured in midgut samples of larvae found feeding on mustard (MT-ctrl), radish (RD-ctrl), and *T. majus* (Tp-ctrl) were significantly higher at *p*≤0.05, as compared to the total midgut proteolytic activities detected in larvae feeding on cauliflower and cabbage. Rubiscolytic activities in these samples were inhibited with STI by at least 20% (Figure 1B), suggesting the presence of STI-sensitive and –insensitive serine proteases active at high alkaline pH.

**Figure 2:**
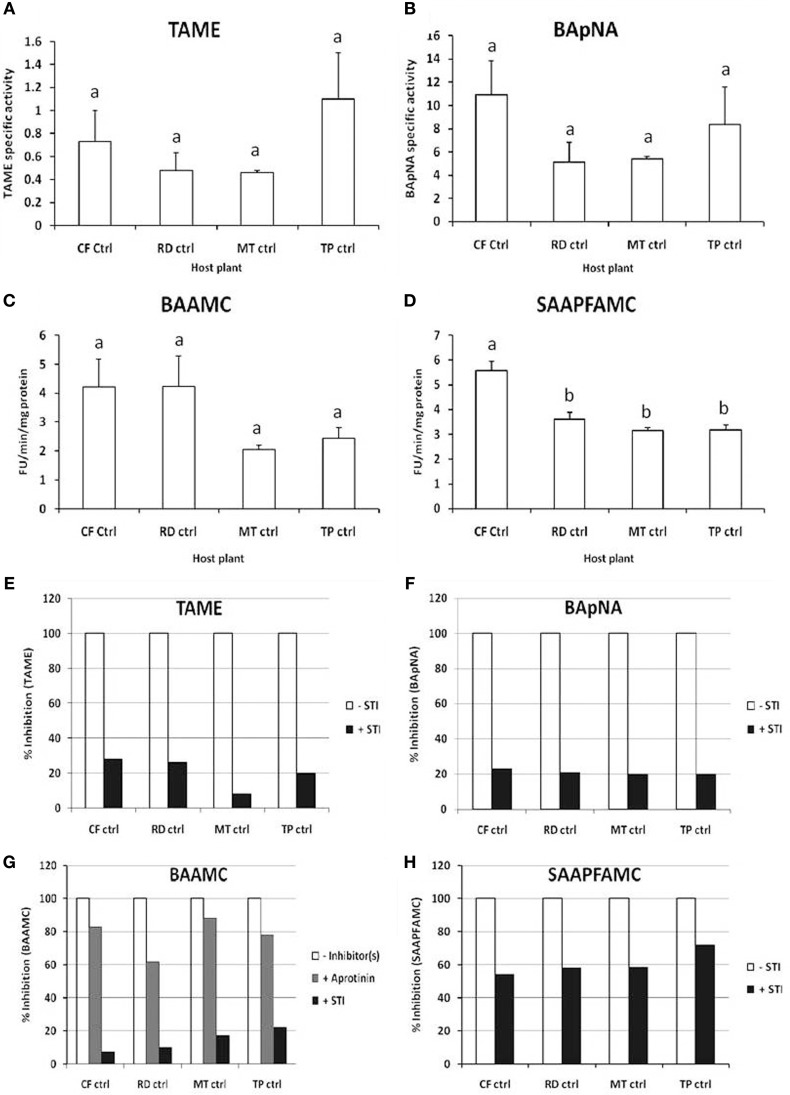
***In vitro* assays for detection of midgut trypsin and chymotrypsin activities in field-collected, fourth instar *P. brassicae* larvae (*n* = 36) feeding on different host plants in the field (CF-ctrl, RD-ctrl, MT-ctrl, and TP-ctrl)**. Substrates used were **(A)** TAME for trypsin activity at pH 8; **(B)** BApNA for trypsin activity at pH 9; **(C)** BAAMC for trypsin activity at pH 10; and **(D)** SAAPFAMC for chymotrypsin activity at pH 10. Specific activity refers to enzyme units hydrolyzed per minute per mg total protein. Fluorogenic assays are shown as Fluorescence Units released per minute per microgram total protein. Bars depict mean ± SE; the same letters in lower case are not statistically significant (*p* ≤ 0.05). Percent inhibition by STI of midgut trypsin and chymotrypsin activities detected in the midgut samples using **(E)** TAME; **(F)** BApNA; **(G)** BAAMC; **(H)** SAAPFAMC substrates are shown. The extent of inhibition is shown relative to proteolytic activities detected in midgut samples (depicted as 100% in absence of STI).

Similar levels of trypsin-like activities were detected in these samples using TAME substrate at pH 8, BApNA substrate at pH 9 and BAAMC substrates at pH 10 (Figures 2A–C). Multiple trypsin substrates were employed as fourth instar *P. brassicae* larvae fed on cauliflower have multiple pH optima for total midgut protease activities detected using protein substrates viz. casein and rRBCL (Bhardwaj et al., 2014). Chymotrypsin activities were detected in the midgut samples using the fluorogenic substrate SAAPFAMC at pH 10 (Figure 2D). The highest midgut chymotrypsin activity was detected in larvae collected from the cauliflower fields.

Less than 25% of the trypsin activities detected in these midgut samples were inhibited by STI (Figures 2E–G). STI was a better inhibitor of the midgut chymotryptic activities, showing greater than 50% inhibition. In case of insects found feeding on *T. majus*, 78% of the midgut chymotrypsin activities were susceptible to STI inhibition (Figure 2H). Trypsin activities detected in these midgut samples were inhibited by at least 80% by Aprotinin, a serine-protease-specific inhibitor, (Figure 2). Chymotrypsin activities were insensitive to TPCK, (a specific inhibitor of mammalian chymotrypsins, not shown). Chymotrypsin activity detected with SLLVYAMC substrate was negligible in all samples tested (not shown). No elastase or cysteine protease activity was detected using SLLVYAMC and MeOSuc-AAPVAMC substrates in the midgut samples in this study (not shown).

**Figure 3:**
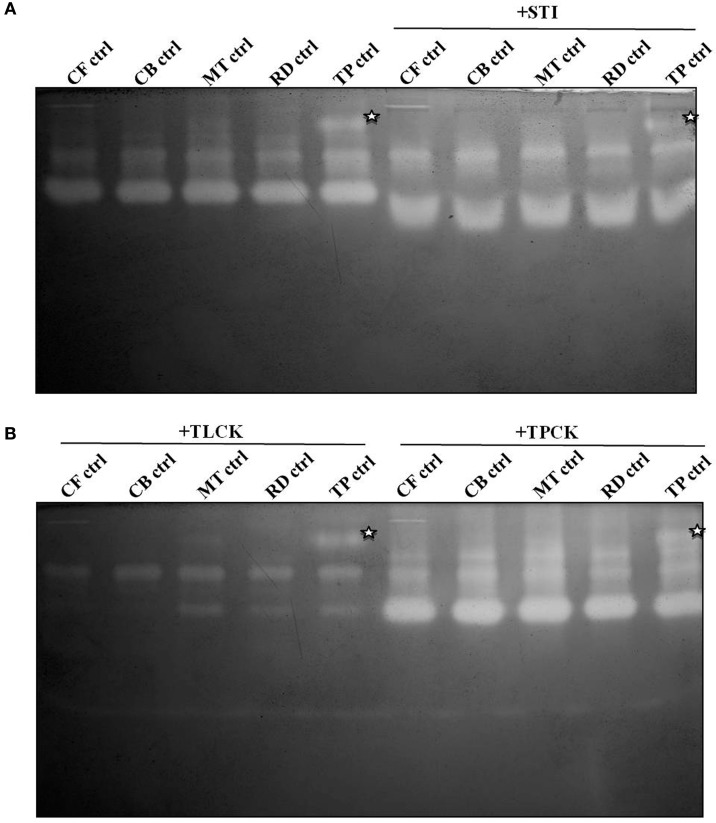
**Gelatin zymograms of midgut samples from field-collected, fourth instar *P. brassicae* found feeding on leaves of cauliflower (CF-ctrl), cabbage (CB-ctrl), mustard (MT-ctrl), radish (RD-ctrl), and *T. majus* (TP-ctrl) in **(A)** absence and presence of STI (5 mg/ml)**. Zymograms of samples **(B)** incubated with inhibitors TLCK (2 mg/ml) and TPCK (2 mg/ml) are also shown. Asterisk denotes activity zone observed only in midgut samples of larvae fed on *T. majus*.

Gelatin zymography with the midgut samples produced two prominent activity zones representing multiple proteases of varied mobility (Figure 3A). In general the activity zones were similar in larval samples collected from fields of different crucifer host plants. An additional, slow-moving activity zone was discernable in larvae collected from *T. majus* plots (Figure 3A, denoted by an asterisk). Figure 3 showed that patterns of activity zones observed in larvae feeding on different diets were similar upon incubation with STI and E-64 (an inhibitor of cysteine proteases). Samples incubated with TLCK, (a trypsin-specific inhibitor) showed lessening in intensity of these activity zones (Figure 3).

### Midgut protease activities detected with casein and rRBCL-Cf substrates in fourth instar larvae transferred from cauliflower to cauliflower and *T. majus* in no-choice, fixed time experiments

**Figure 4:**
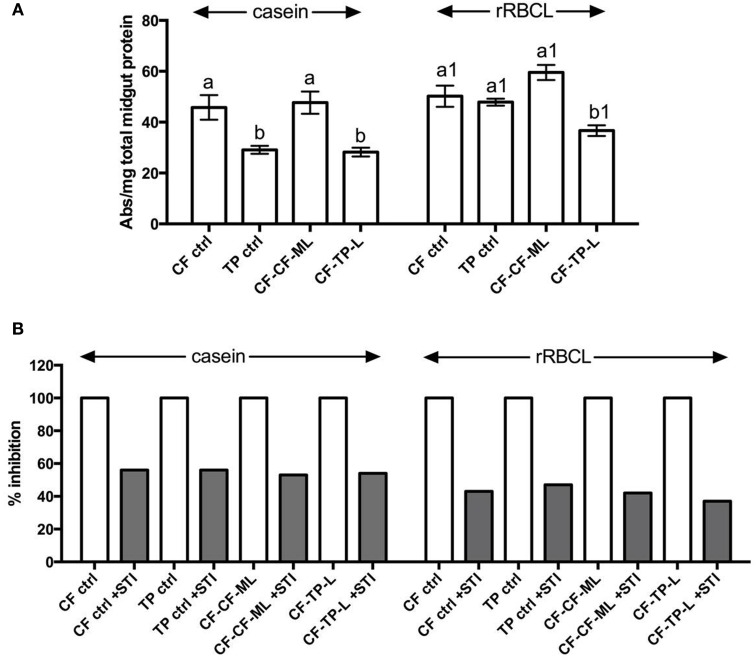
**(A)** Total proteolytic activities detected in midgut samples of fourth instar *P. brassicae* feeding on cauliflower (CF ctrl), *T. majus* (TP ctrl) and from larvae transferred in the lab for 12 h to mature leaves of cauliflower (CF-CF-ML) or leaves of *T. majus*, (CF-TP-L) at pH 10 using casein and recombinant RBCL-Cf as protein substrates. The activities are shown as mean change in absorbance (ABS) ± SE per mg total protein. The same letters in lower case are not statistically significant (*p* ≤ 0.05). **(B)** Percent inhibition of protease activities detected in the same midgut samples in presence of STI at pH 10. The extent of inhibition is shown relative to total proteolytic activities detected (depicted as 100% in absence of STI).

Field-collected fourth instar larvae feeding on mature leaves of cauliflower (CF-ctrl) had similar levels of total midgut proteolytic activities (as measured by digestion of casein and rRBCL-Cf proteins) as larvae transferred to detached, mature leaves of cauliflowers (CF-CF-ML) (Figure 4A). Larvae found feeding on mature leaves of *T. majus* in the garden (TP-ctrl) had lower levels of total midgut protease activities as measured by casein digestion, but equal levels of rRBCL-Cf digestion as compared to larvae found feeding on cauliflower (CF-ctrl), highlighting differences in results with choice of substrate used to measure proteolysis. Larvae transferred from cauliflower to *T. majus* leaves in the lab (CF-TP-L) also had lower levels of midgut protease activities in comparison to the larvae transferred from cauliflower to mature leaves of cauliflower (CF-CF-ML) as detected using casein and rRBCL-Cf substrates. These levels were lower than the field collected larvae found feeding on *T. majus* (TP-ctrl) in the case of rRBCL-Cf substrate.

Approximately 50% of the total midgut protease activities detected in all samples of larvae feeding on cauliflower and *T. majus* were inhibited by STI (Figure 4B). In case of casein substrate, the extents of inhibition of total proteolytic activities by STI ranged from 52 to 55%, while in case of rRBCL-Cf substrate, the extents of inhibition by STI ranged from 40 to 48% of the total midgut proteolytic activities.

### Gelatin zymography of midgut proteases in larvae transferred to, and fed on different tissues of cauliflower and *T. majus* in no-choice, fixed time experiments

**Figure 5:**
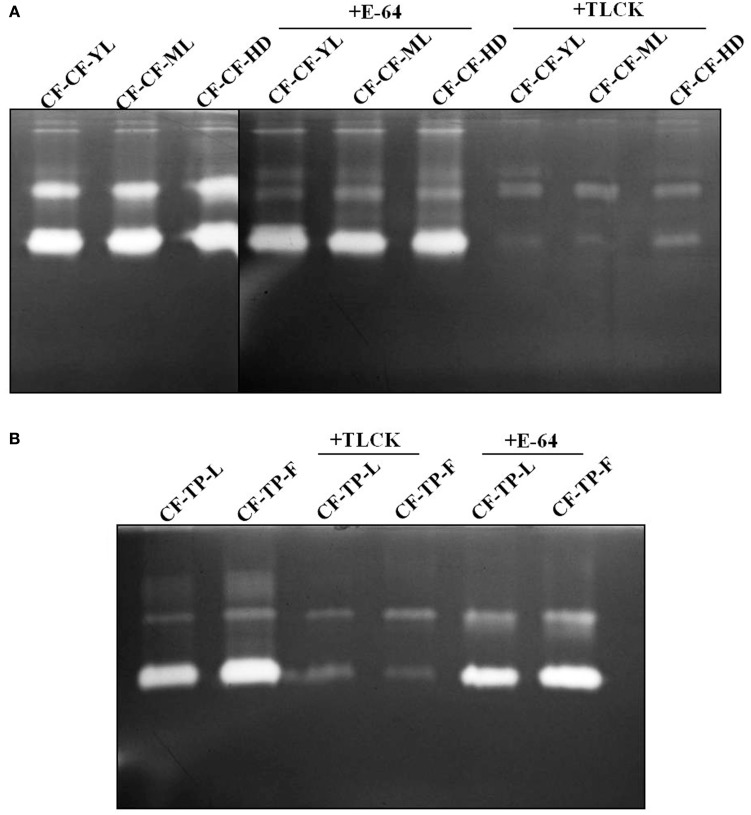
**Gelatin zymograms of *P. brassicae* midgut extracts from fourth instar larvae transferred for 12 h to (A) various tissues of cauliflower (CF-CF-YL, CF-CF-ML, and CF-CF-HD), in presence and absence of E-64 (2 mg/ml) and TLCK (2 mg/ml); or transferred to (B) leaves and flowers of *T. majus* (CF-TP-L and CF-TP-F), in presence and absence of TLCK (2 mg/ml) and E-64 (2 mg/ml)**.

Two prominent activity zones representing midgut proteases were detected by gelatin zymography in larvae transferred from mature leaves of cauliflower to detached cauliflower young leaves (CF-CF-YL), cauliflower mature leaves (CF-CF-ML), and cauliflower head (CF-CF-HD) tissues for 12 h (Figure 5A). A similar pattern along with a faint activity zone of slow mobility was observed for larvae transferred from mature leaves of cauliflower to detached leaves and flowers of *T. majus* (CF-TP-L and CF-TP-F) for 12 h (Figure 5B). Incubation of midgut extracts with E-64 (a diagnostic inhibitor of cysteine proteases) showed no differences in patterns of activity zones obtained among larvae feeding on different tissues of each host plant (Figure 5). Incubation with TLCK produced a reduction in the intensity of gelatinolytic activities, but neither complete inhibition nor change in patterns of activity zones were discerned for larvae feeding on different tissues of cauliflower and *T. majus*.

### Midgut serine proteases in fourth-instar larvae transferred from cauliflower to various detached, plant tissues of cauliflower and *T. majus* in no-choice, fixed time experiments

**Figure 6:**
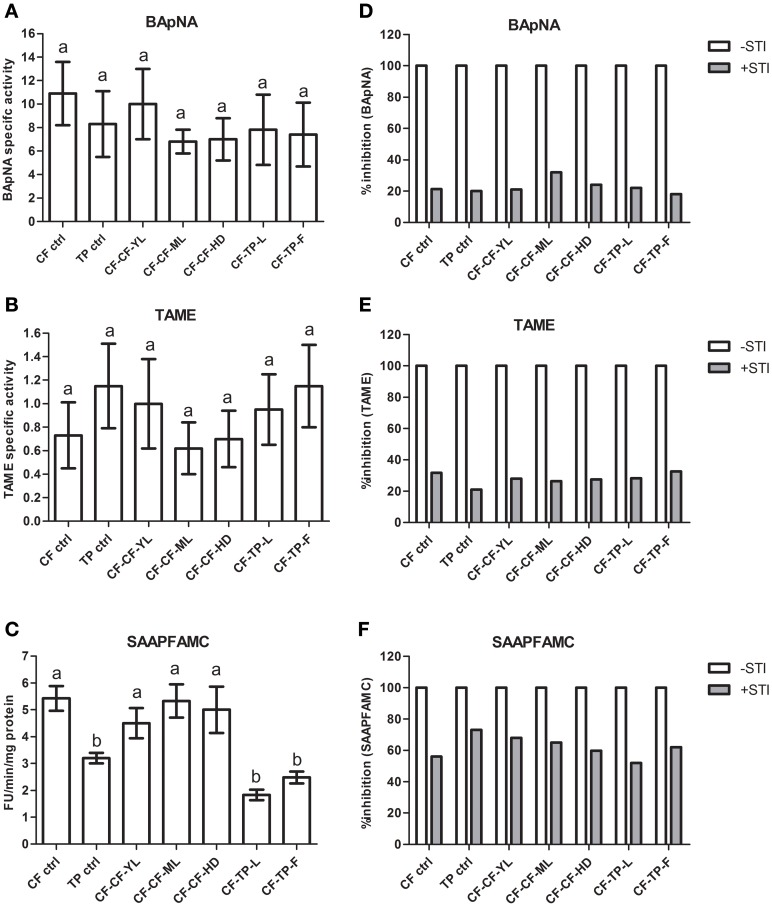
**Midgut trypsin and chymotrypsin activities detected using various synthetic substrates (A: BApNA; B: TAME; C: SAAPFAMC) in field-collected fourth instar *P. brassicae* larvae (*n* = 36) found feeding on cauliflower (CF-ctrl) and *T. majus* (TP-ctrl)**. Also shown are midgut serine protease activities in larvae transferred in the lab to detached mature leaves (ML), young leaves (YL) and curd/head (HD) of cauliflowers (CF) as well as leaves (L) and flowers (F) of *T. majus*. Specific activity refers to enzyme units hydrolyzed per minute per mg total protein. Bars depict mean ± SE; the same letters in lowercase are not statistically significant (*p* ≤ 0.05). Percent inhibition by STI of midgut trypsin and chymotrypsin activities detected in the midgut samples using **(D)** BApNA; **(E)** TAME; **(F)** SAAPFAMC substrates are shown. The extent of inhibition is shown relative to proteolytic activities detected in midgut samples (depicted as 100% in absence of STI).

Figures 6A–C compared midgut serine protease activities in fourth instar larvae collected from mature leaves of cauliflower growing in the field (CF-ctrl) and *T. majus* in the garden (TP-ctrl) as well as larvae transferred from cauliflower to various aerial tissues of these host plants for 12 h in the lab. Midgut trypsin activities using the substrates BApNA (at pH 10, Figure 6A) and TAME (at pH 8, Figure 6B) were not significantly different (*p* ≤ 0.05) between these samples.

In contrast, chymotrypsin activities detected in larvae fed on cauliflower using the substrate SAAPFAMC (pH 10) differed significantly (*p* ≤ 0.05) from chymotrypsin activities detected in larvae feeding on *T. majus* (Figure 6C). Chymotrypsin activities in the samples of larvae fed on *T. majus* collected from the garden (TP-ctrl), larvae transferred from cauliflower mature leaves to *T. majus* leaves (CF-TP-L) and flowers (CF-TP-F) were lower than those detected in larvae fed on cauliflower collected from the field (CF-ctrl), larvae transferred from cauliflower to young leaves (CF-CF-YL), mature leaves (CF-CF-ML), and head (CF-CF-HD) of cauliflower in the lab (Figure 6C). Levels of chymotrypsin activities in midgut samples of larvae feeding on different tissues of the same host plant collected from the field, garden or transferred in the lab were similar.

Figures 6D–F shows percentage inhibition of midgut trypsin and chymotrypsin activities detected using TAME (at pH 8), BApNA (at pH 10), and SAAPFAMC (at pH 10) substrates by STI. Trypsin activities in all midgut samples were inhibited by approximately 20%. Thus, most of the trypsin activities detected in these samples were STI-insensitive. In contrast, the midgut chymotrypsin activities detected in these samples were inhibited by at least 50% by STI (Figure 6F). Trypsin activities detected in these midgut samples were inhibited by at least 80% by Aprotinin while the chymotrypsin activities were insensitive to TPCK (not shown).

### Comparison of nutritional indices among larvae transferred from cauliflower to various detached tissues of cauliflower and *T. majus* in no-choice, fixed time experiments

**Figure 7:**
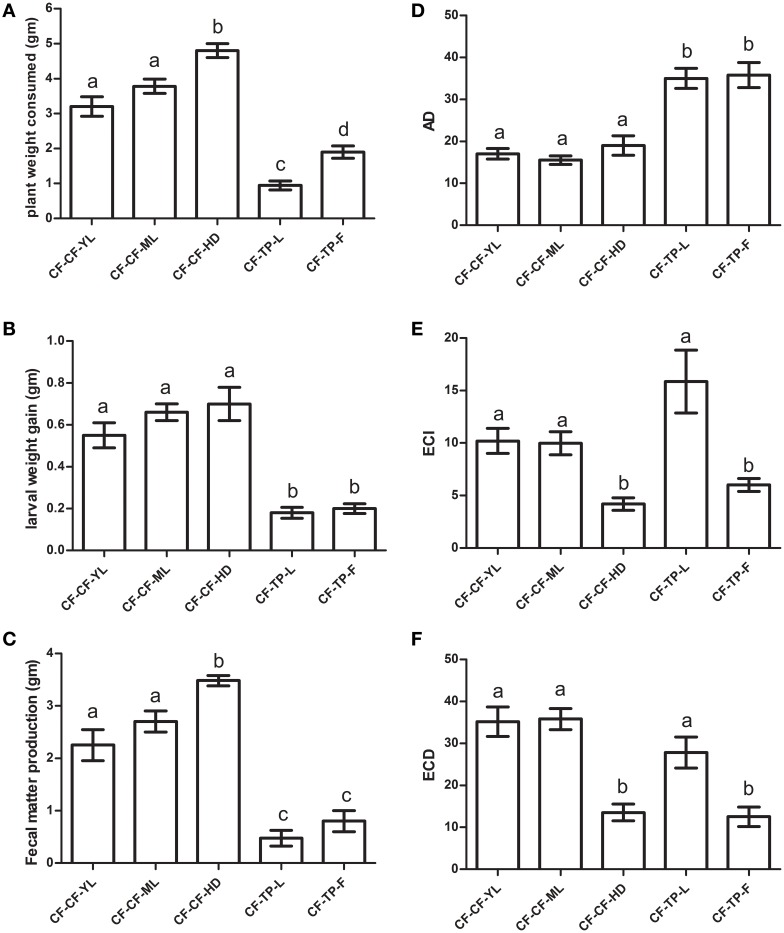
**Comparison of (A) plant weight consumed, (B) larval weight gained (WGL), (C) fecal matter produced, (D) AD (or Approximate Digestibility), (E) ECI (or Efficiency of Conversion of Ingested Matter), and (F) ECD (or Efficiency of conversion of Digested matter) of fourth instar *P. brassicae* larvae (*n* = 36) collected from CF fields and transferred to detached tissues (ML, YL, HD/F) of cauliflower (CF) and *T. majus* (TP) for 12 h in no-choice experiments in the laboratory**. The bars represents mean ± SE; the same letters indicate no statistically significant difference (*p* ≤ 0.05).

Approximate digestibility measures (AD) among fourth instar larvae transferred to different tissues of cauliflower (mature leaves or CF-CF-ML, young leaves or CF-CF-YL and head or CF-CF-HD) were similar, but lower than those transferred to leaves (CF-TP-L) and flowers (CF-TP-F) of *T. majus* (Figure 7D). Despite larvae consuming more tissues of cauliflower heads than young or mature leaves (Figure 7A), AD measures for all three tissues of cauliflower were similar because the amounts of fecal matter produced by larvae feeding on the head tissues of cauliflower were also higher than that of larvae feeding on leaf tissues of cauliflower (Figure 7C). Measures of efficiency of converison of ingested food (ECI) and efficiency of conversion of digested food (ECD) varied for larvae to leaf and floral tissues of *T. majus*. Larvae gained equal weight on both tissues, produced similar amounts of fecal matter, but consumed more food when feeding on flowers (Figures 7B,C). This was reflected in significant differences among ingested tissues of *T. majus* for measures of ECI and ECD (Figures 7E,F). Larvae consumed more food and produced more fecal matter when shifted to head tissues of cauliflower (CF-CF-HD), as a result of which ECI and ECD for larvae transferred to HD tissues were significantly different from larvae transferred to YL and ML tissues. Interestingly, the larvae performed equally well in term of ECD when in the transfers to CF-CF-YL; CF-CF-ML and CF-TP-L.

Significant positive correlations were observed between larval weight gained by insects transferred to different detached tissues of cauliflower and their midgut trypsin and chymotrypsin activities measured using BApNA substrate at pH 9 (*R*^2^ = 0.641, *N* = 25, *p* ≤ 0.05) and SAAPFAMC substrate at pH 10 (*R*^2^ = 0.493, *N* = 25, *p* ≤ 0.05). However, non-significant weak to nil correlations (at *p* ≤ 0.05) were observed between midgut serine protease activities and weight gained by larvae transferred to detached tissues of *T. majus*. Similar trend in results were observed with larvae collected as neonates from cauliflower fields and reared in the lab up to the 4th instar on cauliflower ML tissues prior to transfer experiments (not shown). It should be mentioned here that YL, ML or HD/F tissues compared here (CF vs. TP) are obviously not equivalent in the taxonomic or chemical sense. They have been used as a criteria for distinguishing available tissues that *P. brassicae* larvae can feed.

### *In vitro* assays for *P. brassicae* midgut trypsin activities and inhibition using CfTI and TpTI trypsin inhibitors identified from cauliflower and *T. majus*

**Figure 8:**
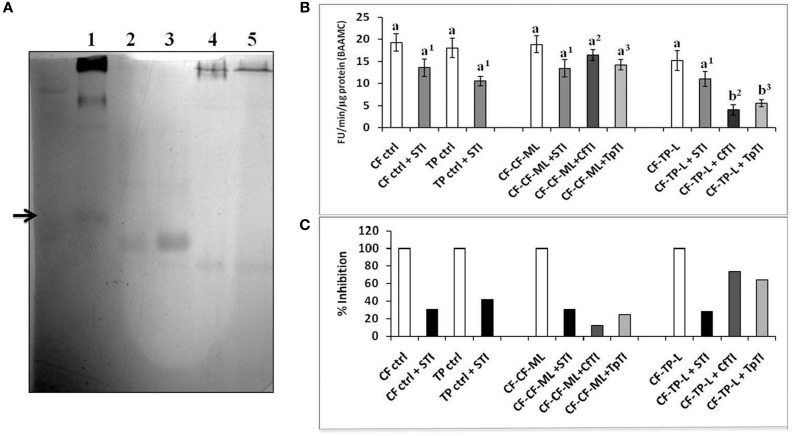
**(A)** Reverse zymogram using Bovine Trypsin (2 mg/ml), to detect putative trypsin inhibitors from un-induced cauliflower and *T. majus* leaves (lanes 2 and 4, 10 μg) and induced, *P*. *brassicae*-attacked cauliflower (CfTI) and *T. majus* (TpTI) leaves (lanes 3 and 5, 10 μg). Lane 1 shows a standard legume trypsin inhibitor. **(B)** Fluorogenic assays with BAAMC substrate at pH 10 using midgut samples from field collected fourth instar *P. brassicae* larvae found feeding on mature leaves of cauliflower (CF-ctrl), leaves of *T. majus* (TP-ctrl) and transferred for 12 h in the laboratory to detached mature leaves of cauliflower (CF-CF-ML) and detached leaves of *T. majus* (CF-TP-L). Midgut trypsin activities are shown as Fluorescence Units released per minute per microgram total protein. Bars depict mean ± SE; the same letters and numbers in upper case are not statistically significant (*p* ≤ 0.05). **(C)** Percent inhibition by STI (black bars); CfTI (gray bars), and TpTI (light gray bars). The extent of inhibition is shown relative to proteolytic activities detected in midgut samples (depicted as 100% in absence of inhibitor).

Putative trypsin inhibitors were isolated using affinity column chromatography with (bovine) trypsin agarose from leaves of cauliflower and *T. majus* plants (i) on which fourth instar larvae were feeding (induced) and (ii) plants that were insect-free and healthy (un-induced). The purified proteins readily interacted with bovine trypsin in reverse zymography (Figure 8A, Figure S3). LC-ESI-MS of both proteins from cauliflower showed the most significant match to a wound-induced Kunitz Trypsin inhibitor from wild cabbage, *Brassica oleracea* (BoPI), of protein mass = 23534 (Figure S4, Table S1). The number of peptides matched between these proteins were 5, covering 31% of the BoPI sequence (GenBank accession # T14442, Table S2). LC-ESI-MS data of both proteins from *T. majus* indicated homology to various proteins, including a thaumatin-like protein (*p* ≤ 0.05, Table S1). Two matching peptides were identified from GenBank accession # Q5ND92-ACTDE, a thaumatin-like protein from Kiwi, with 20% sequence coverage (Figure S4, Table S2). Bovine trypsin activity detected with the fluorogenic substrate, BAAMC, was strongly inhibited by CfTI (97%) and TpTI (95%) at pH 8. Both CfTI and TPTI were ineffective against bovine chymotrypsin activities detected using the fluorogenic substrate, SAAPFAMC (not shown).

CfTI inhibited the midgut trypsin activity in CF-CF-ML samples by only 20% (Figures 8B,C). This result indicated that most trypsin-like components of the larval midgut proteases were not susceptible to CfTI, and probably represented trypsin inhibitor-insensitive enzymes “adapted” to the presence of dietary inhibitors including CfTI. In contrast, midgut trypsins in samples of larvae fed on detached leaves of *T. majus* (CF-TP-L) were inhibited by about 82% with CfTI indicating midgut proteases susceptible to CfTI in larvae transferred from cauliflower to detached, leaves of *T*. *majus* (Figures 8B,C). At least 28% of the midgut trypsin activities detected using BAAMC substrate in all samples was inhibited by STI, with the highest extent of inhibition (42%) observed in TP control samples. Like CfTI, TpTI was a poor inhibitor of midgut trypsin activities (only 20%) detected in larvae transferred to mature leaves of cauliflower (CF-CF-ML samples), but a better inhibitor (70%) inhibitor of midgut trypsins in larvae transferred to *T. majus* leaves or CF-TP-L samples (Figure 8C). These results clearly indicated that even though the levels of midgut trypsins were similar in these samples (Figure 8B), their susceptibilities to endogenous (putative) trypsin inhibitors like CfTI and TpTI were different. These results also showed that despite CfTI and TpTI being different proteins (lack of similarity in LC-ESI-MS results), their abilities to inhibit bovine trypsin and midgut trypsin activities in CF-CF-ML and CF-TP-L samples were similar.

## Discussion

Alkaline midgut serine proteases (like trypsins) were first reported from *P. brassicae* in 1960s by the classical work of Marguerite Lecadet and her associates (Lecadet and Dedonder, 1967). Larval midgut trypsins and chymotrypsins have been implicated in ecological adaptations of *P. rapae* (Broadway, 1989a). Luminal and membrane-bound serine proteases were recently reported in *P. brassicae* larvae feeding on cabbage (Zibaee, 2012). In this study, total rubiscolytic activities were compared in fourth instar larvae feeding on various crucifers and *T. majus*. More protease activity was detected in larvae collected from radish, mustard and *T. majus* as compared to those found feeding on cauliflower and cabbage), suggesting that either more proteases were present in these samples or that the substrate (native Rubisco) was more accessible to gut hydrolases. It is also possible that ingestion of elevated levels of endogenous PIs in these host plants resulted in increased production of larval midgut proteases.

An additional proteolytic activity zone was observed in zymograms of gut samples from field-collected fourth instar feeding on *T. majus*, indicating differential expression of gut proteases in contrast to larvae feeding on cauliflower. Subsequent experiments addressed midgut proteases in larvae feeding on these two host plants. Larvae transferred from mature leaves in cauliflower field to detached mature leaves of cauliflower contained statistically similar levels of proteases (determined using casein and recombinant RBCL-Cf substrates), suggesting that comparison of protease levels found in gut tissues from larvae fed various plant tissues may be a valid assessment for digestive physiological conditions occurring when larvae move to and feed on alternate hosts plants. In contrast, total proteolytic activities in larvae transferred from cauliflower mature leaves to *T. majus* leaves had lower amounts of total proteases detected using the above-mentioned protein substrates. These results also suggested that the time frame of these experiments (12 h) was sufficient to detect inherent physiological plasticity in expressions of midgut proteases in *P. brassicae* larvae when faced with host plant shift as fourth instar larvae.

Proteases active at alkaline pH usually belong to the serine protease class of hydrolases (Berenbaum, 1980). Multiple pH optima exist for gut proteases from pierid larvae (Broadway, 1989a; Zibaee, 2012; Bhardwaj et al., 2014). The pH optima of midgut proteases detected in field-collected fourth instar larvae transferred to cauliflower and *T. majus* using casein, and recombinant RBCL substrates was found to be 8–10 (Figure S1). Hence proteolytic activities in gut samples of larvae fed tissues from different hosts were examined using multiple substrates at various pH. Trypsin activities measured at pH 8–10 were similar in all samples, but chymotrypsin levels measured at pH 10 were significantly lower in larvae fed on radish, mustard and *T. majus* as compared to those fed on cauliflower and cabbage. Midgut trypsin activities were similar among larvae feeding on various tissues of cauliflower and *T. majus*. However, midgut chymotrypsin activities were generally reduced in case of larvae transferred to *T. majus* leaves and flowers, indicating quantitative down-regulation of corresponding enzymes in larvae feeding on an alternate host species. Our results suggested that chymotrypsin activities in these larvae transferred to an alternate host plant species shift toward the “optimum” (as indicated by larvae feeding on *that* host plant species in the field). Differential chymotrypsin levels in response to feeding on different species of host plants have been reported from Saturniid larvae of *A. assamensis* (Saikia et al., 2011). Reduced chymotrypsin activities have also been observed in *P. brassicae* larvae feeding on diets containing antifeedants like lectins (Zibaee et al., 2014). The reasons for differential expression of chymotrypsins are not immediately clear.

Plant protease inhibitors are usually end-products of well-characterized plant defense response pathways to herbivory (Ryan, 1990; Koiwa et al., 1997). They are expressed differentially in time and space in crucifers along with secondary metabolites including glucosinolates (Broadway and Colvin, 1992; Cipollini et al., 2003). Foliar trypsin inhibitor activities have been reported in several cultivated and wild crucifers (Broadway, 1989b). Genes encoding Kunitz trypsin inhibitors with high sequence similarity to homologs from *A. thaliana* are present in the crucifers studied here and *T. majus* (Kumar, 2009; GenBank accessions EU126810–EU126815). In this study, CfTI was isolated from cauliflower leaves on which *P. brassicae* larvae were feeding. LC-ESI-MS results indicated that CfTI resembled BoPI, a trypsin inhibitor reported from wild cabbage (Williams et al., 1997). Dietary BoPI reduced larval growth of *Heliothis virescens* but not cauliflower pests that were presumably adapted to the inhibitor (Pulliam et al., 2001). In our study CfTI was ineffective (at the concentration tested) in inhibiting the proteases of larvae fed caulilfower, indicating the presence of CfTI-insensitive midgut trypsins. In contrast, CfTI strongly inhibited trypsin activities in larvae transferred from cauliflower to *T. majus*, indicating qualitatively different expression of digestive trypsins upon shift to a new host plant type.

Substantial (bovine) trypsin inhibitory activities have been reported previously in leaf extracts of *T. majus* (Broadway, 1989b). In our study, TpTI (isolated from leaves of *T. majus* fed upon by *P. brassicae*) almost completely inhibited bovine trypsin activity. TpTI poorly inhibited midgut trypsins in larvae fed on cauliflower but, like CfTI, was a more effective inhibitor of trypsins in larvae feeding on *T. majus*. Similar results were verfied using rRBCL-Cf protein substrate (not shown). LC-ESI-MS results indicated that among inhibitors, TpTI resembled thaumatin-like family of proteins (Ascenzi et al., 1999). Dual specificity trypsin/amylase inhibitors with strong sequence similarity to thaumatin-like proteins have been reported from maize (Richardson et al., 1987; Ryan, 1990). Multiple protease inhibitors including Kunitz serine protease inhibitor, RTI/MTI-2 and Thaumatin families are present in crucifers including *A. thaliana* (Ascenzi et al., 1999; Ruoppolo et al., 2000; Clauss and Mitchell-Olds, 2004). Further work is necessary to fully characterize defense-related inhibitors in *T. majus*. In our study, both induced and uninduced cauliflower plants contained CfTI, while induced and uninduced *T. majus* plants contained TpTI. The presence of plant protease inhibitors in plants showing little/no evidence of herbivory has been attributed to baseline constitutive expression of corresponding genes (Zavala and Baldwin, 2004).

It is well-known that adaptation of lepidopteran insects to dietary PPIs involves expression of larval midgut serine proteases that are insensitive to the ingested PPI (Broadway, 1995; Jongsma et al., 1995; Mazumdar-Leighton and Broadway, 2001a,b; Terra and Ferreira, 2012). Poor inhibition of midgut trypsins of *P. rapae* by STI has been reported previously (Broadway, 1989a, 1996). Our study also reports STI-insensitive midgut trypsins in *P. brassicae* larvae feeding on several crucifers, as well as different tissues of cauliflower and *T. majus*. Interestingly, midgut chymotrypsins in *P. brassicae* larvae (whose levels responded quantitatively to ingested host plant type) were more sensitive to STI, suggesting different regulation of trypsins and chymotrypsins in this insect. STI is known to inhibit both trypsin and chymotrypsin activities in lepidopteran larvae (Mazumdar-Leighton and Broadway, 2001a). Finally, trypsins and chymotrypsins are major proteases, but not the only hydrolases involved in proteolysis of ingested proteins in *P. brassicae* (Zibaee, 2012). Further work is necessary to examine the nature and role of gut enzymes during digestion of different host plants. The complexity of physiological responses to a host plant shift evoked in fourth instar larvae of *P. brassicae* is unlikely to be due to just trypsin inhibitors

Acceptability of a host plant by a herbivore is manifested primarily in the ability of larvae to digest ingested plant tissues, convert food material into essential nutrients required for growth and increased body weight (Slansky and Feeny, 1977; Broadway and Duffey, 1986b; Woods and Kingsolver, 1999; Joern et al., 2012). The extent of digestion is known to be affected by chemistry and nutritive quality of the ingested food, larval growth stage, energy expended by the insect, and rate of assimilation into body tissue (David and Gardiner, 1962; Schoonhoven and Meerman, 1978; Kaplan et al., 2014). Various reports have demonstrated that *P. brassicae* larvae develop at different rates on different host plant types (Ansari et al., 2012; Mehrkhou et al., 2012; Zibaee et al., 2014). We showed here that fourth instar *P. brassicae* larvae transferred from cauliflower to *T. majus* consumed less tissues, excreted less, weighed less and had higher AD values after 12 h than their counterparts transferred to various tissues of cauliflower. Approximate digestibility in larvae feeding on different species of host plants is a measure likely influenced by levels and efficacy of proteolytic digestion by trypsins, chymotrypins, and other gut hydrolases. Among cauliflower tissues, ECI and ECD measures were variable for larvae feeding on cauliflower heads. The head tissues of cauliflowers are known to influence feeding measures and nutritional parameters differently from the leaves (Sharma and Gupta, 2009; Hasan and Ansari, 2010; Ansari et al., 2012).

Pierids can compensate for low nutritional quality of food ingested by increasing food consumption and efficiency of food utilization, producing results resembling larvae fed high nutritional foods (Slansky and Feeny, 1977; Chen et al., 2004). Increased feeding is energetically more expensive and incurs costs like sustained production of digestive enzymes. In this study, larvae transferred from mature leaves of cauliflower to *T. majus* consumed more flowers than foliage as a result of which ECI and ECD measures were lower for larvae feeding on *T. majus* flowers. The ability of early larval instars of *P. brassica* to feed on flowers of host plants like *Brassica nigra* has been reported earlier (Smallegange et al., 2007; Lucas-Barbosa et al., 2014). Indeed growth (and nitrogen use efficiency) of *P. brassicae* on *T. majus* is reported to be less robust attributed to the glucosinolate glucotropaeolin and/or poorly digestible nitrogenous host plant material (Slansky and Feeny, 1977). Metabolic costs have been held responsible for significant differences in ECI and ECD values of fifth instar *P. brassicae* transferred from cabbage to *T. majus*, despite showing comparable AD values (Schoonhoven and Meerman, 1978). Interestingly, in our study ECD measures were similar for larvae transferred to various tissues of cauliflower and leaf tissues of *T. majus*. Comparable ECD measures on foliage of *T. majus* may reflect the ability of the insect to utilize this host species.

Significant positive correlations were observed between midgut trypsin and chymotrypsin activities; larval weight gain and ECD measures estimated for larvae transferred to different tissues of cauliflower (Kumar, 2009). However, no clear correlations were discernable between midgut serine protease activities and larval weight gains in the case of transfers to *T. majus*. Absence of correlations between increased proteolytic activities and larval weight gain may reflect (i) transient expression of proteases involved in adaptation to protease inhibitors present in a new food; (ii) physiological processes that do not contribute to body growth *per se* (immune responses or hydrolysis of non-PI anti-feedants), or (iii) proteases being less important for digestion/nutrition than enzymes critical for breakdown of ingested carbohydrates or lipids. Intake of balanced amounts of carbohydrates, lipids and proteins are integral for assimilation of macro- and micronutrients, optimal growth, nutrition and development of locusts (Raubenheimer and Simpson, 1998, 2003). Routes of cross talk between physiological mechanisms in *P. brassicae* that govern efficient metabolism of ingested proteins, carbohydrates and lipids during adaptation to a new host plant are not known and require future studies.

Various reports have suggested that *T. majus* is a sub-optimal host for *P. brassicae* (Ma, 1972; Schoonhoven and Meerman, 1978; Kaushal and Vats, 1983; Metspalu et al., 2003). Nevertheless, *P. brassicae* females oviposit on and larvae feed prolifically on *T. majus* in the field. In order to observe the context in which our study was carried out, neonates from egg clusters oviposited on cauliflower were transferrred to enclosed, intact plants of cauliflower and *T. majus*. When reared on *T. majus*, fourth instar larvae originating from egg clusters laid on cauliflower weighed less (at *p* ≤ 0.05) than larvae reared on cauliflowers (Figure S2). While the numbers of larvae reaching pupation were not significantly different, adult eclosions were significantly higher on cauliflower than *T. majus*. Larvae reared on *T. majus* took longer to reach maturity than larvae reared on cauliflower (Figure S2). Similar results have been reported for duration of larval instar, numbers of pupating insects and pupal weight in *P. brassicae* feeding on cauliflower vs. *T. majus* (Metspalu et al., 2003). Variable eclosion rates have been reported for *P. brassicae* feeding on different crucifers under field and lab conditions (Ali and Rizvi, 2007). Lengthening of any stadium of development can make insect populations susceptible to predators (Bernays and Weiss, 1996; Benrey and Denno, 1997). Both predatory wasps and *Cotesia glomerata* infestations take a heavy toll on *P. brassicae* larvae found late in the season in cauliflower fields in North India (Kumar, 2009).

Delaying larval growth and development can be a potential pest management strategy for controlling crucifer pests. Crucifer pests are also targets of proposed transgenic plant protection strategies that target the insect midgut, including *Bacillus thuringiensis* (Bt) endotoxins genes and genes for inhibitors of midgut digestive enzymes (Sharma et al., 2000; Earle et al., 2004; Shelton et al., 2009; Schlüter et al., 2010; Zibaee, 2012; Zibaee et al., 2014). This study was undertaken to contribute knowledge toward development of environmentally safe, sustainable strategies for control of *P. brassicae* using protease inhibitors for the region. Studies with its digestive physiology involving midgut serine proteases, and nutritional indices for alternate host plant transfers suggest that *P. brassicae* is a versatile pest that can feed on all aerial parts of its host plants. However, shift to alternate host plants in the lab suggest that metabolic costs may be incurred. Further work is needed to understand the effects of alternate host plant utilization on life history traits and susceptibility of larvae to parasites and predators. Recurrence of this pest may be stymied (but not prevented) by inter-cropping with non-host non-crucifers. This may be done spatially or temporally (to dissuade over-wintering of *P. brassicae* larvae in these fields or their vicinity). Transgenic strategies may also be attempted. However, tissue-specific promoters for expression of transgenes should not be used. As an alternate to Bt transgenes or in conjunction, transgenes encoding chymotrypsin inhibitors should be explored.

## Author Contributions

All authors contributed equally to project design and experimentation, data analyses and interpretations, as well as finalization of paper.

### Conflict of interest statement

The authors declare that the research was conducted in the absence of any commercial or financial relationships that could be construed as a potential conflict of interest.
