# Supplementary material for: Midgut serine proteases and alternative host plant utilization in Pieris brassicae L
Source: Front Physiol. 2015 Mar 31;6:95. doi: 10.3389/fphys.2015.00095 (PMC4379908; doi:10.3389/fphys.2015.00095)
Supplement: Supplementary file 1 [file Presentation1.PDF]

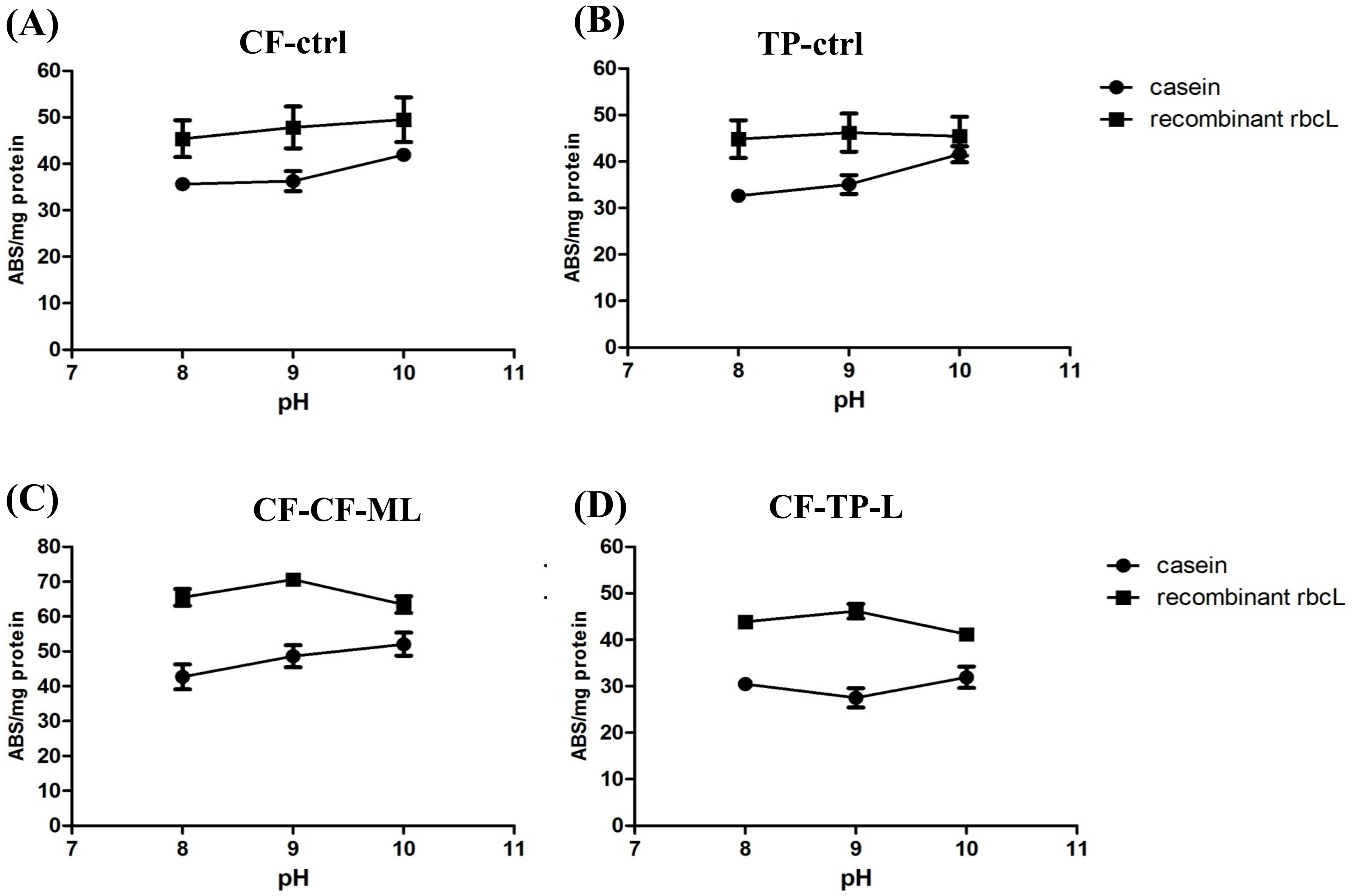

Figure S1

Figure S1: Differences in pH optima of total proteolytic activities detected in midgut samples of fourth instar *P. brassicae* detected using casein and recombinant RBCL-Cf as protein substrates. The activities are shown as mean change in absorbance (ABS) ± SE per mg total protein. Midgut samples were obtained from field-collected larvae found feeding on (**A**) mature leaves of cauliflower, CF-ctrl; (**B**) *T. majus* leaves, TP-ctrl, and from larvae transferred in the lab for 12 hours to (**C**) mature leaves of cauliflower, CF-CF-ML and (**D**) leaves of *T. majus*, CF-TP-L.

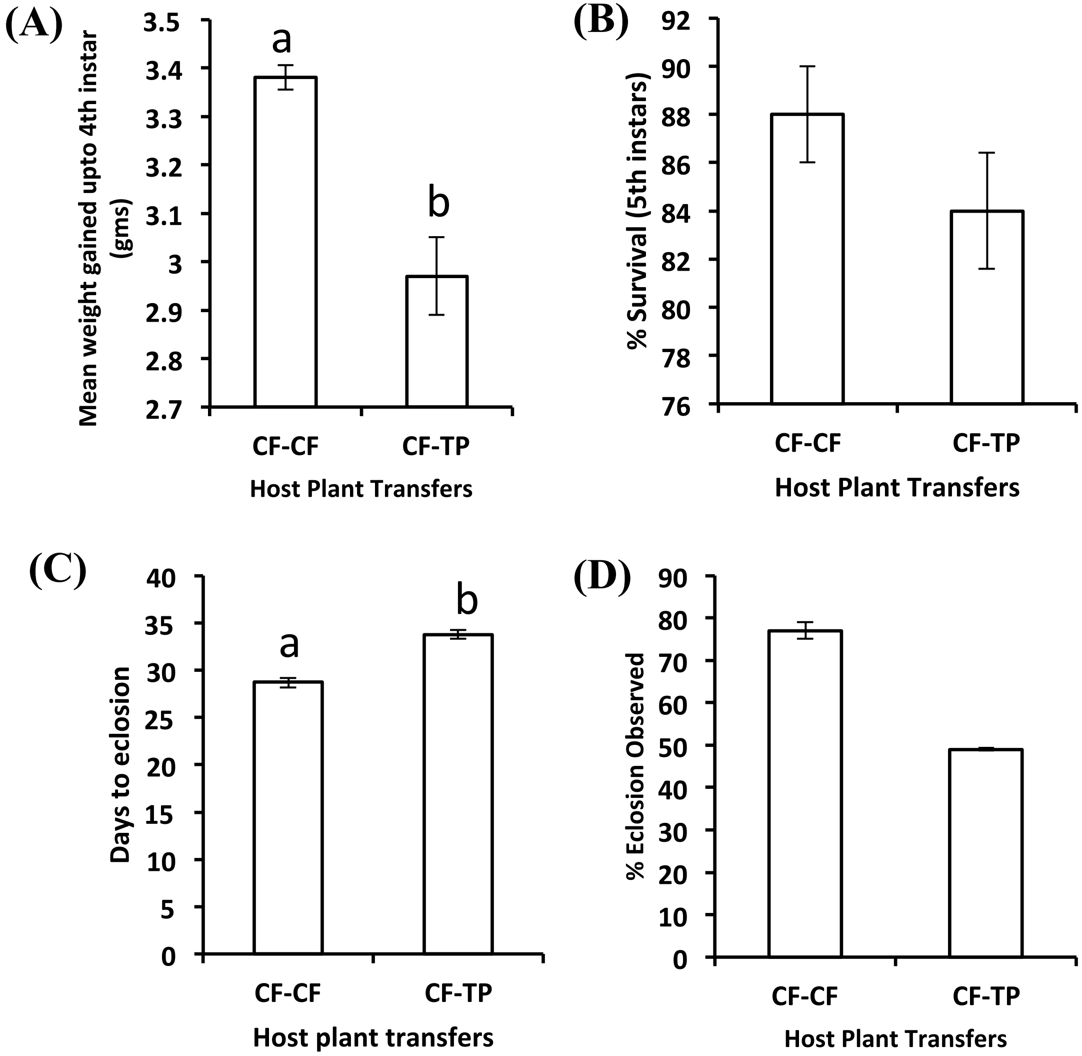

Figure S2

Figure S2: Comparison of field-collected larvae of *P. brassicae* transferred as neonates (n=50) from mature leaves of cauliflowers to mesh-enclosed intact plants of cauliflower (CF-CF) and *T. majus* (CF-TP) for traits (A) Mean weight gained up to fourth instar; (B) percentage survival up to pupation; (C) Days to eclosion; and (D) Percentage eclosion. Different letters in lower case are not statistically significant ($p \leq 0.05$).

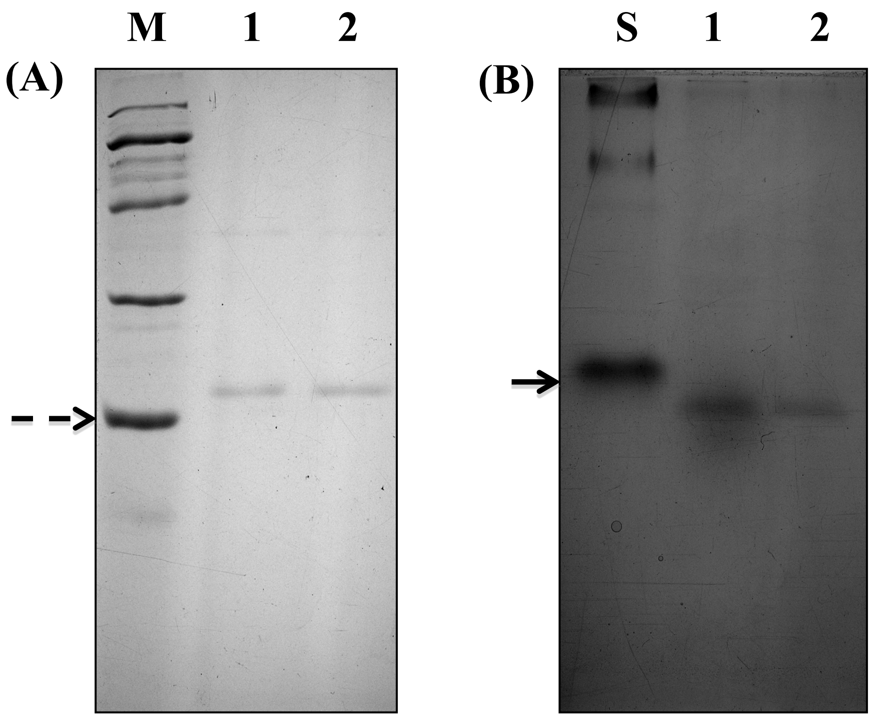

Figure S3

Figure S3: (**A**) A 12.5% SDS-PAGE showing bovine trypsin-agarose purified, putative trypsin inhibitors from insect-free, un-induced mature leaves (lane 1) and *P. brassicae*-attacked induced mature leaves or CfTI (lane 2) from cauliflower. Lane M shows pre-stained SDS-PAGE size marker (Broad range, Sigma #161-0318). (**B**) A reverse zymogram using Bovine Trypsin (2 mg/ml) detected putative trypsin inhibitors from un-induced mature leaves (lane 1, 10 µg) and *P. brassicae*-attacked, induced mature leaves or CfTI (lane 2, 10 µg) from cauliflower. Lane S shows a standard legume trypsin inhibitor. Dashed and Bold Arrows indicate STI used as a standard.

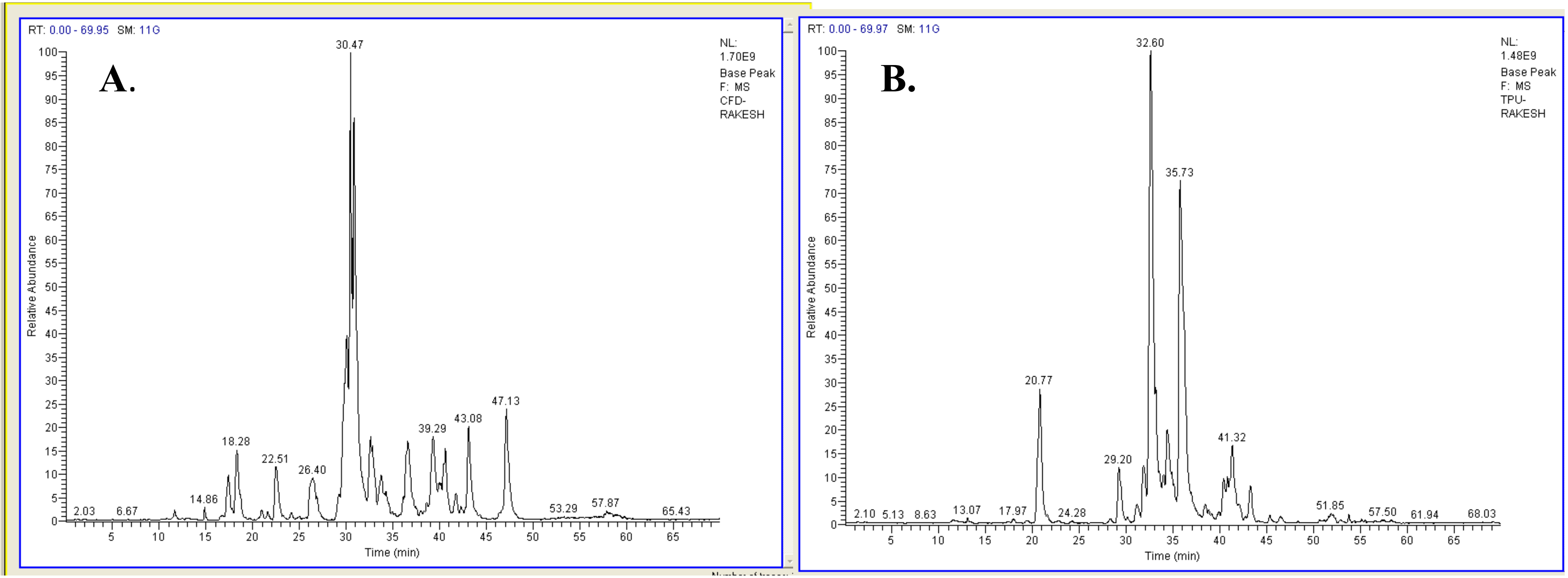

Figure S4

Figure S4: Spectra obtained from LC-ESI-MS of putative trypsin inhibitors: (**A**) CfTI isolated from cauliflower (*B. oleracea* var. botrytis) mature leaf tissues, and (**B**) TpTI from nasturtium (*T. majus*) mature leaf tissues performed by TCGA Ltd., Delhi, India.

# Hit	Accession #	Protein Name	Protein Score	# Peptides matched
List of top 5 significant protein hits for CfTI				
**1**	**T14442**	**Trypsin inhibitor precursor -wild cabbage**	**309**	**5**
2	AAF02131	ATAC009918 NID: - *Arabidopsis thaliana*	184	8
3	Q676X3_HYAOR	2-cys peroxiredoxin-like protein (fragment) *Hyacinthus orientalis* (Common Hyacinth)	142	6
4	CAA71503	AT2CPBAS1- NID: - *Arabidopsis thaliana*	129	6
5	Q9SQJ4_BRACM	2-Cys peroxiredoxin, *Brassica campestris* (Field mustard)	111	6
List of top 5 significant protein hits for TpTI				
1	T03685	Probable superoxide dismutase precursor, chloroplast, rice	83	3
2	Q6ER94-ORYSA	Putative thioredoxin peroxidase, chl, *Oryza sativa* (japonica cultivar – group)	68	3
**3**	**ABQ42566**	**Thaumatin-like protein, *Actinidia deliciosa* (Kiwi)**	**58**	**2**
4	Q676X3_HYAOR	2-cys peroxiredoxin-like protein (fragment) *Hyacinthus orientalis* (Common Hyacinth)	57	2
5	AAB51566	ATU75188 NID: - *Arabidopsis thaliana*	49	3

Table S1: A list of proteins detected ($p<0.05$) by LC-ESI-MS of CfTI and TpTI performed by TCGA Inc., New Delhi, India. Parameters adopted for Mascot searches were as follows:- Type of search: MS/MS Ion search; Enzyme: Trypsin; Fixed modifications: Carbamido-methylation (C); Variable modifications: Oxidation (M); Protein mass: Unrestricted; Peptide mass tolerance = ±2 Da; Fragment mass tolerance = ±0.8 Da; Maximum missed cleavage = 1; Instrument Type = ESI-TRAP. Proteins 'scores' were derived from ion scores as a non-probabilistic basis for ranking protein hits. Proteins with known inhibitory activity towards bovine trypsin are shown in bold and elaborated in Table S2.

Observed	Mr (expt)	Mr (calc)	Delta	# Miss	Score*	Peptide Sequence
(**A**) For CfTI sample, peptides identified from Accession# T14442 (Trypsin inhibitor precursor from wild cabbage) :						
608.2100	607.2027	607.3329	-0.1302	0	20	K.LNAYNK.F
769.2800	768.2727	768.470	-0.1443	0	27	R.SFFQIK.K
744.6136	1487.2127	1486.7667	0.4460	0	52	R.VGFVPEEENLNIK.M
945.1536	1888.2927	1887.9326	0.3601	0	82	R.SLFIAAGPKPEAGGEDSSR.S
1424.7486	2847.4827	2847.3149	0.1678	0	40	K.MDVEPTICAQSAYWWVTPAPSPWR.S
(**B**) For TpTI sample, peptides identified from Accession# Q5ND92-ACTDE (Thaumatin-like protein, Kiwi) :						
711.5186	1421.0227	1420.6228	0.4	0	58	R.APGGCNNPCTVFK.T
621.1600	620.1527	620.2992	-0.1465	0	12	K.VVCFP.-

Table S2: A list of Mascot (www.mascot.com) peptide summary reports related to plant protease inhibitors obtained from LC-ESI-MS for (**A**) CfTI and (**B**) TpTI samples. 'Observed' refers to experimental m/z values obtained from a selected peak list. 'Mr (expt)' refers to the expected molecular mass from the observed m/z value. 'Mr (calc)' refers to the molecular mass of the matched peptide. 'Delta' is the difference between expected and calculated values. '# Miss' refers to expected number of missed cleavages. Ion scores* were defined as -10*log (P) where P is the probability that the observed match is a random event. Individual random scores >44 indicated identity or extensive homology ($p<0.05$). One-letter amino acid codes denote the 'peptide sequences'. Amino acids that flank each tryptic fragment are shown next to a period (.). A dash (-) denotes a peptide at the carboxyl terminus.
